# Definitive chemoradiation in locally advanced inoperable esophageal cancer patients – Retrospective analysis of different chemotherapy regimens in a tertiary cancer centre

**Published:** 2021-10-30

**Authors:** Aswin Nagarajan, Begum Yesmin Nureja, Ramya Ravichandar, Rama Ranganathan

**Affiliations:** ^1^Department of Radiation Oncology, Cancer Institute, Adyar, Chennai, Tamil Nadu, India; ^2^Department of Pharmacology, Sree Balaji Medical College, Chennai, Tamil Nadu, India; ^3^Department of Epidemiology, Biostatistics and Cancer Registry, Cancer Institute, Adyar, Chennai, Tamil Nadu, India

**Keywords:** chemoradiation, paclitaxel, carboplatin, cisplatin, overall survival

## Abstract

**Background and Aim:**

Definitive chemoradiation (dCRT) is the standard treatment for locally advanced inoperable esophageal cancer. The aim of this study is to analyze the effect of dCRT combined with paclitaxel and carboplatin (TC) against cisplatin (CDDP) with radiation.

**Methods:**

The study population included patients with locally advanced inoperable esophageal cancer seeking treatment at our center from March 2013 to December 2017. Case records from 66 patients were extracted. The toxicity profile of patients who received TC or CDDP was reported and analyzed. A Chi-square test and students t-test were used to analyze the categorical, and the continuous variables, respectively. The KaplanMeier method was used to estimate the survival probability. A log-rank test was applied to compare the survival differences between the two groups.

**Results:**

The overall survival (OS) did not differ at 3 years between the TC and CDDP (p = 0.286). The median survival duration was 13 months for CDDP and 18 months for TC. The toxicity profile like emesis (93% CDDP vs. 25% TC), neutropenia (79% CDDP vs. 13% TC), thrombocytopenia (10% CDDP vs. 17% TC) and dyselectrolytemia (71% CDDP vs. 8% TC) were compared between the two treatment groups and found to be more in CDDP group.

**Conclusion:**

The treatment of patients with locally advanced esophageal carcinoma with dCRT and TC showed an improved toxicity profile, but similar OS compared to CDDP. Applying dCRT with TC could be an alternate regimen for locally advanced inoperable esophageal cancer patients.

**Relevance for Patients:**

Concurrent chemoradiation with TC regimen can be considered as an alternative for cisplatin as it shows equivalent survival and reduced toxicity profile

## 1. Introduction

Esophageal cancer contributes to 6% of the total gastrointestinal tumors. It ranks as the fourth most common cause for cancer-related deaths in India, and eighth worldwide [1]. The incidence increases with age, reaching the peak starting at the age of 60 [2]. The symptoms of esophageal cancer are vague, leading to a late presentation at the clinic. Almost half the patients present either at locally advanced stage or with metastasis and thereby the cure rate is as low as 15% [3].

Definitive chemoradiation (dCRT) is the preferred surgery for patients with esophageal cancer who are either unresectable or medically unfit for surgery. Cisplatin (CDDP) and 5 fluorouracil (5FU) along with radiation are the most commonly used standard regimen for unresectable esophageal cancer. Patients unfit for CDDP/5FU or with extensive comorbidity, receive another regimen with paclitaxel and carboplatin (TC). Neoadjuvant chemoradiation followed by surgery study (CROSS) showed improved overall survival (OS) in patients with resectable esophageal or esophagogastric junctional cancer [4]. The Radiation Therapy Oncology Group (RTOG) 85-01 trial showed that chemoradiation with CDDP and 5FU improved the 5-year survival rate compared to radiotherapy alone in patients who were not undergoing surgery [5]. For both regimens, a radiotherapy dose 50.4 Gy is used. So far, no study showed the superiority of one regimen over the other in terms of OS.

The aim is to study the effect of radiotherapy with TC versus CDDP in definitive setting, with respect to OS and toxicity profile.

## 2. Methods

### 2.1. Study population

The case records of patients with esophageal cancer treated in our institute from March 2013 to December 2017 were extracted. All patients gave informed consent during the study period. The study was approved by the Cancer Institute Ethics Committee (IEC/2020/June 08).

Patients with locally advanced disease, according to the RTOG 85-1 trial, deemed inoperable by surgical oncologists were included. The inclusion criteria were: invasion of the trachea, adherence to the pericardium, the encasement of the aorta by more than 180° visualized by imaging, the patient’s general condition, the presence of comorbidity. Patients were excluded when: operable by the surgical team during the study period, received the CROSS protocol, had non-regional node/distant metastasis, or received palliative treatment.

The patient’s evaluation consisted of basic investigations, upper gastrointestinal endoscopy, biopsy and 18F-fluorodeoxyglucose positron emission tomography (FDG-PET), and computerized tomography (CT). Bronchoscopy was performed when the tumor was tethered to either main stem bronchus or trachea according to the images.

The chemotherapy regimen consisted of two groups:

1) CDDP regimen (n = 42): Either 3 weekly CDDP at a dose of 75 mg/m^2^ on days 1, 22, and 43 or weekly CDDP at a dose of 40 mg/m^2^ on days 1, 8, 15, 22, 29 and 36 and
2) Paclitaxel (50 mg/m^2^) and carboplatin (AUC 2) regimen (n = 24): on days 1, 8, 15, 22, 29, and 36.

Patients with Performance Status of 1 and body surface area (BSA) >1.5 m^2^ (n = 25, 38%) received 3-weekly CDDP. Patients with a BSA between 1.3 m^2^ and 1.5 m^2^ (n = 17, 26%) received weekly CDDP. Patients with BSA <1.3m^2^(n = 24, 36%) received TC.

Our institutional policy is to give a single agent-CDDP in definitive setting. Since many of our patients developed high morbidity, the compliance decreased with an increased mortality rate. Therefore, the Multidisciplinary Board decided to proceed only with CDDP along with radiotherapy in definitive setting. Even though the standard protocol followed worldwide for CDDP regime is CDDP + 5FU [5]. The combination of capecitabine with CDDP and radiotherapy was not routinely practiced in the study period (2013 – 2017).

### 2.2. Radiotherapy

Radiotherapy planning was carried out either by 2D or 3D. In 2D planning, the patient swallowed barium for localization of the primary tumor and was simulated in the supine position. The gross tumor volume (GTV), defined as the macroscopic primary tumor and regional lymph node metastases, was reconstructed using all available information derived from endoscopy, CT, and FDG-PET. A margin of 5cm cephalo-caudally and 2cm transversely was given from the GTV, and patients were treated with 3 field technique.

The patients treated with 3D conformal technique underwent a planning CT in which GTV was localized. The clinical target volume was obtained by adding a 3cm margin in the cephalo-caudal direction, and a 1cm margin in the transversal plane. A margin of 0.5 – 1 cm was used around the pathological lymph nodes. The planning target volume was obtained by adding a 2 cm margin cephalo-caudally and a 1cm margin in transverse plane, as per our institutional policy. The mean dose delivered was 5410 cGy in the CDDP arm and 5264 cGy in the TC arm (range 42 – 60 Gy) using 6MV Photons with the fractionation schedule of 180 – 200 cGy/day.

### 2.3. Patient follow up

Patients were advised for regular follow-up according to the hospital guidelines at 6 weeks after completion of the treatment, then every month for the first 6 months, followed by once every 2 months for the next 18 months, subsequently once every 3 months for the next 12 months, followed by once every 6 months for the next 24 months, and thereafter annually until death.

### 2.4. Outcomes

Toxicity, disease-free survival (DFS), and OS were the outcomes of interest. Toxicity was measured according to the Common Terminology Criteria for Adverse Events (CTCAE 5). DFS was determined from the starting date of treatment to documented date of first recurrence or death from any cause. OS was defined as the time interval between the starting date of the chemoradiation and documentation of the day of death or last follow-up, whichever was earlier.

### 2.5. Statistical analysis

The continuous variables in the study were age and BSA. The categorical variables were gender, location of the tumor, histology, tumor status (T), and node status (N) (UICC 8^th^ edition). The continuous variables were analyzed using the student’s t-test, and the categorical variables by using the chi-square test. OS and DFS rates were estimated using the Kaplan–Meier method and compared using the log-rank test. An alpha-value of 5% was used as threshold for statistical significance. Statistical analysis was done using SPSS Version 20 [6].

## 3. Results

### 3.1. Patient characteristics

We extracted 66 case records from patients with locally advanced inoperable esophageal cancer treated at our center during the study period.

The patient’s characteristics are reported in Table 1. The mean age was 49.6 (standard deviation, SD: 9.50) years in the CDDP group and 51.67 (SD 10.04) years in the TC group (p = 0.407). There was no difference in gender between either intervention groups (p = 0.376). The most prevalent cancer site was the middle thoracic esophagus (56%) in the CDDP group, and 45% in the TC group (p = 0.910). The most common histology was squamous cell carcinoma in the CDDP (42 squamous cell carcinoma) as well as in the TC (22 squamous cell carcinoma vs. 2 adenocarcinoma) arms (p = 0.128). There was no significant difference in squamous cell carcinoma prevalence. The performance status of 2 was found in 4 patients (9%) in the CDDP group and 9 patients (37%) in the TC group. Other patients in both groups had the performance status of 1 (91% in CDDP vs. 63% in TC (p = 0.009). The presence of comorbidity in the CDDP group and the TC group included diabetes mellitus type 2 (p = 0.144) and systemic hypertension (p = 0.550). The T/N factor groups were T4 (60% in CDDP arm vs. 50% in TC arm), T4aN0-2 (33% in CDDP arm vs. 46% in TC arm), and T3-N1 (7% in CDDP arm vs. 4% in TC arm). There was no difference between the two groups on tumor characteristics (p = 0.597). The mean radiotherapy dose was 54.12 ± 2.76 Gy in the CDDP arm and 52.64 ± 2.15 Gy in the TC arm and significantly higher in the CDDP arm (p = 0.029).

**Table 1:** Patient characteristics

Variable	CDDP (n=42)	TC (n=24)	p value
Age (mean±SD)	49.6±9.50	51.67±10.04	0.407
Gender			
Male	24 (57%)	11 (46%)	0.376
Female	18 (43%)	13 (54%)	
Performance status			
1	38	15	0.009*
2	4	9	
Stage Group			
T3 N1^@^	3 (7%)	1 (4%)	0.577
T4a N0-2	14 (33%)	11 (46%)	
T4b N0-3	25 (60%)	12 (50%)	
Histology			
Squamous cell carcinoma	42 (100%)	22 (92%)	0.128
Adenocarcinoma	0 (0%)	2 (8%)	
Grade			
II	14 (33%)	10 (42%)	0.498
III	28 (67%)	14 (58%)	
Site			
Cervical	4 (9%)	3 (13%)	0.910
Upper thoracic	5 (11%)	3 (13%)	
Middle thoracic	23 (56%)	11 (45%)	
Lower thoracic	10 (24%)	7 (29%)	
Diabetes mellitus			
Yes	6 (14%)	7 (29%)	0.144
No	36 (86%)	17 (71%)	
Systemic hypertension			
Yes	10 (24%)	4 (17%)	0.550
No	32 (76%)	20 (83%)	
Radiotherapy dose (mean±SD)	54.12±2.76^#^	52.64±2.15	0.029*

@ Four patients were stage III (T3N1) have received concurrent chemoradiation due to factors like elderly age, ischemic heart disease and poor lung compliance.

# Two of the patients in the CDDP arm received low radiotherapy dose - one patient received 42Gy (poor tolerance) and another 48.6Gy (reason not clearly mentioned).

* Significant

### 3.2. Treatment outcomes

**Figure 1:**
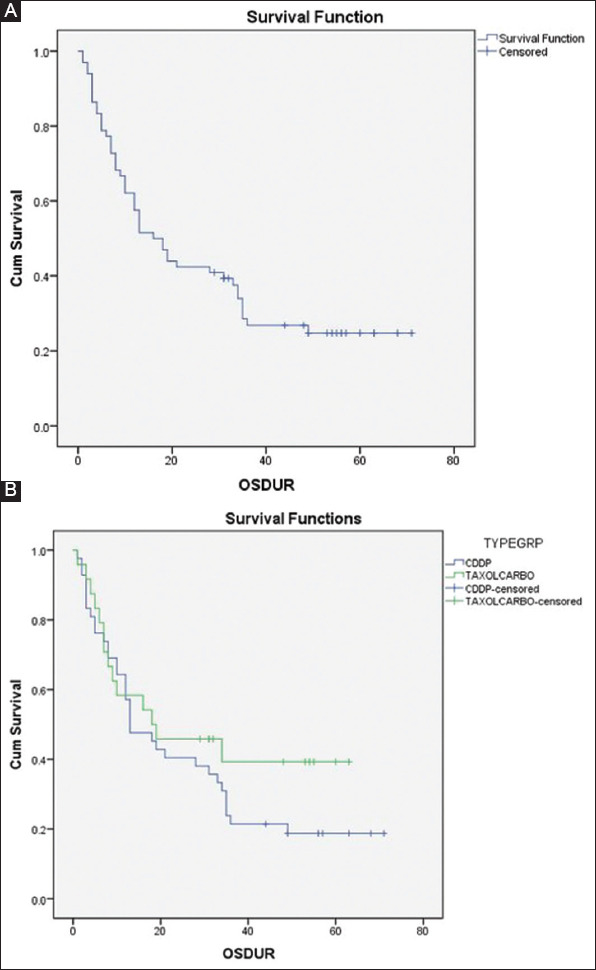
(A and B) The overall survival between the cisplatin and paclitaxel and carboplatin group.

**Figure 2:**
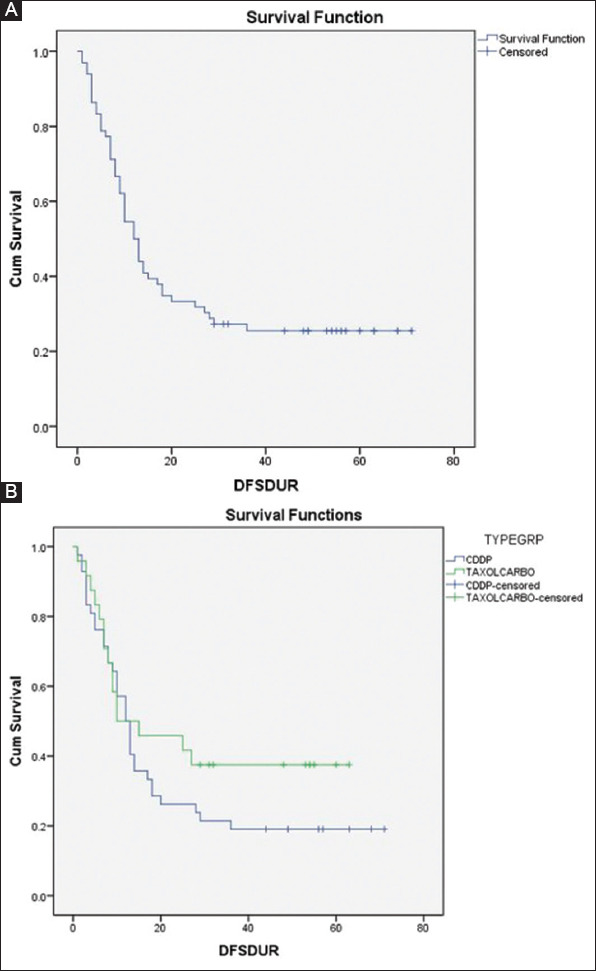
(A and B) The disease-free survival between the cisplatin and paclitaxel and carboplatin group.

Complete response on follow-up endoscopic evaluation was n = 25 (60%) in the CDDP arm, and n = 16 (67%) in the TC arm. The median OS was 13 months in the CDDP arm and 18 months in the TC arm (p = 0.286). No statistically significant difference in OS was reported between both groups (Figure 1A and B and Figure 2A and B).

The CDDP regimen was associated with both hematological and non-hematological toxicity when compared with the TC regimen (Table 2). The non-hematological toxicities dyselectrolytemia and emesis were significantly higher in the CDDP group (p = 0.001). Among the 39 patients who had emesis in CDDP arm, 13 patients had grade I, 21 patients had grade II, and 5 patients had grade III emesis. All six patients in the TC arm had grade II emesis. The exact grading of dyselectrolytemia in both the arms was not reported in the extracted case records and therefore excluded from the final analysis. In the CDDP arm, 11 patients had grade III esophagitis, and the rest had grade II esophagitis. In the TC arm, 7 patients had grade III esophagitis, and the remaining patients had grade II esophagitis (p = 0.782).

**Table 2:** Toxicity outcome

Toxicity outcome	CDDP (n=42)	TC (n=24)	p value
Dyselectrolytemia			
Yes	30 (71%)	2 (8%)	0.001*
No	12 (29%)	22 (92%)	
Emesis			
Yes	39 (93%)	6 (25%)	0.001*
No	3 (7%)	18 (75%)	
Esophagitis			
Grade II	31 (74%)	17 (71%)	0.782
Grade III	11 (26%)	7 (29%)	
Neutropenia			
Yes	33 (79%)	3 (13%)	0.001*
No	9 (21%)	21 (87%)	
Thrombocytopenia			
Yes	4 (10%)	4 (17%)	0.392
No	38 (90%)	20 (83%)	

* significant

Neutropenia cases were significantly higher in the CDDP group (n = 33, 93%) compared to the TC group (n = 3, 13%) (p = 0.001), wherefrom 17 had grade I, 10 had grade II, and 6 had grade III neutropenia. In the TC group, all 3 cases had grade I neutropenia without any febrile neutropenia. No difference in thrombocytopenia was found (p = 0.392).

## 4. Discussion

Our study showed that radiotherapy given along either with CDDP or TC in the definitive setting resulted in similar OS outcome. The follow-up endoscopy was similar in both arms. When the toxicity profiles were compared, CDDP resulted in more toxicities (both hematological and non-hematological). To the best of our knowledge, there were no studies in the literature comparing single agent CDDP with TC for dCRT setting in locally advanced inoperable esophageal cancers. However, alterative regimens were reported in the literature on toxicity and OS.

### 4.1. OS

Multiple studies reported on the effect of carboplatin with paclitaxel, CDDP, CDDP/5FU, and TC on OS in dCRT for esophagus cancer [4,7-13]. None found a difference in OS when any of the regimens were compared. The latter is consistent with our reported finding of no difference in OS outcomes. Only one study reported improved OS in CDDP/5FU when compared to TC [14]. Nonetheless, the study population was relatively small, and they had localized carcinoma of the esophagus, and gastroesophageal junction. Our study included the patients with locally advanced esophageal cancer, which showed similar results in OS in both the regimens. A phase II study on the use of dCRT with TC reported an overall 3-year survival rate of 60% in a small group of esophageal cancer patients with locally advanced disease [15].

### 4.2. Toxicity

We found an improved toxicity profile in the TC arm compared to the CCDP arm. This result is consistent with studies published earlier also reporting on lower toxicity rates and higher compliance to TC [4,8,9,13,16]. One study reported higher rates of toxicity in the TC arm, but also elevated treatment withdrawal [10]. The latter was attributed to the patient selection criteria [10]. In the study of Edmunds *et al*. (2017), the TC chemotherapy was associated with improved pathological response in patients undergoing all three treatment modalities [8]. Furthermore, this study also reported a decrease in weight loss in all patients of the TC arm compared to CDDP/5-FU chemotherapy [8]. The study of Blom *et al*. (2014) compared CDDP/5-FU and TC in the neoadjuvant setting in esophageal cancer patients and showed comparable overall toxicity [13].

Chen *et al*. (2019) compared the OS of patients treated with CDDP/5FU and paclitaxel/5FU. There was no difference between both interventions in the randomized control trial [11]. We found that hematological and non-hematological toxicities were higher with CDDP regimen, which was consistent with earlier studies [9,16]. When we compared the toxicity profile of the two regimens, the CDDP arm had more grade III emesis, esophagitis and neutropenia when compared to the TC arm. There was no grade IV toxicity in either arms. Only 1 patient in the CDDP arm had febrile neutropenia, because Since more patients developed grade III esophagitis, the need for Ryle’s tube dependency was higher on the CDDP arm. Although there is an increased risk of aspiration pneumonitis due to Ryle’s tube dependency, none of our patients developed this complication. With the above discussion, we can conclude that although CDDP and TC chemotherapy regimens have similar OS, one can choose TC regimen in the definitive setting with radiotherapy in view of its favorable toxicity profile.

### 4.3. Study limitations

Our study is limited by the population size, retrospective design, and chemotherapy regimen. The multidisciplinary board of the hospital allowed to provide only CDDP (either weekly or 3 weekly) without 5FU in dCRT setting. This decision was made due to poor compliance with 5FU. Therefore, exact comparison of the efficacy of the chemotherapy regimen was not possible. End-to-end comparison of CDDP and TC was difficult due to the single-dose agent of CDDP. Another important feature is that patients unfit for CDDP received TC. Hence, both the regimens were not completely comparable. The available data allowed us to compare both regimens on OS and toxicity. A randomized clinical trial with a large power is needed to prove the benefit of one regimen over the other with respect to compliance, survival, and toxicity.

## 5. Conclusion

Chemoradiation with TC or CDDP in esophageal carcinoma has the same OS. Regarding improved toxicity profile, TC was the preferred regimen. dCRT with TC could be made as an alternative regimen in patients with locally advanced, inoperable carcinoma esophagus.

### Conflicts of Interest

The authors declare no conflict of interest.
